# Special Issue: “Canine Genetics 2”

**DOI:** 10.3390/genes14101930

**Published:** 2023-10-12

**Authors:** Tosso Leeb

**Affiliations:** Institute of Genetics, Vetsuisse Faculty, University of Bern, 3001 Bern, Switzerland; tosso.leeb@unibe.ch; Tel.: +41-31-684-23-26

**Keywords:** dog, *Canis lupus familiaris*, wild canids, inherited disease, precision medicine, genetic diversity

## Abstract

Wolves were the first animal species to become domesticated by humans, approximately 30,000–50,000 years ago. Human-directed dog breeding over thousands of generations has generated more than 350 recognized breeds displaying surprisingly different phenotypes with respect to morphology, behavior and disease predispositions. The domestication of wolves and the subsequent breeding of dogs can be viewed as one of humankind’s oldest and largest genetic experiments and provides us with unique opportunities for research. Dogs have not only become human’s best friend but were also described as geneticists’ best friend in a past issue of Science. In recognition of the importance of canine genetics, this Special Issue, entitled “Canine Genetics 2”, was compiled. It represents a sequel to the former Special Issue “Canine Genetics”, which was published in 2019. During the last 15 years, the canine community has heavily relied on a reference genome derived from the female Boxer Tasha. “Canine Genetics 2” includes an article describing a greatly improved version of this important community resource. This Special Issue further contains several reports related to monogenic or complex inherited diseases in dogs. Finally, important aspects of wild canid research, genetic diversity in different populations and canine morphology were investigated.

Dogs (*Canis lupus familiaris*) represent the species with the greatest phenotypic variability among mammals [1]. Modern dogs are the result of continuous or intermittent human selection that has extended over hundreds or even thousands of generations. Nowadays, we still have access to dogs’ wild ancestors, the wolf (*Canis lupus*); feral populations of descendants from early domesticated dogs, such as the Australian Dingo; so-called village dogs; various kinds of random-bred and mixed-breed dogs; and finally purebred dogs, which are bred within strictly isolated populations. All purebred dog populations underwent very tight bottlenecks at breed formation, which took place between the 18th century and today [2,3]. Domestication and subsequent dog breeding has formed unique population structures that greatly facilitate the identification of causal genetic variants underlying heritable phenotypes [3,4]. Traits of interest in canine research include their hugely variable morphology, but also inherited diseases that often closely resemble homologous diseases in humans. Heritable disease phenotypes in dogs comprise monogenic and complex diseases, including hereditary predispositions to cancer. Consequently, the dog has been recognized as an important and valuable model for biomedical research [3,5]. The importance of canine genetic research and the very successful Special Issue entitled “Canine Genetics” from the year 2019 prompted the production of a sequel, “Canine Genetics 2”, which contains 21 original articles that were published between December 2020 and April 2022. In the following paragraphs, a very brief, thematically structured overview of this research will be given.

*Resources for Canine Genetic Research*

The release of a genome reference assembly from a female Boxer called Tasha in 2005 represented an important milestone in canine genetic research [6]. This assembly was generated via Sanger sequencing of bacterial clones and, consequently, had numerous gaps in GC-rich regions, including the promoters and first exons of many genes. Nonetheless, it has been a central resource for the canine community for many years. This Special Issue contains an article reporting on a greatly improved genome assembly of Tasha that has a hugely improved contiguity [7]. In the meantime, the new assembly was also annotated by NCBI and is available on the NCBI Genome Viewer [8].

*Canine Morphology*

Dog genomes have approximately 13 times more retrogene insertions than human genomes [9]. In this Special Issue, Bannasch et al. identified two dog breeds that segregate the well-known *FGF4* retrogene insertions on chromosomes 12 and 18, which cause chondrodystrophy and chondrodysplasia, respectively. Using standardized body measurements on a large number of dogs with all possible genotypes, the authors report the individual effects of the different *FGF4* retrogene insertions on skeletal morphology [10]. Two further studies investigated aspects of coat color variation. The first of these confirms that *MC1R* and *MITF* are associated with the quantitative amount of white spotting in Beagles [11]. The other investigated the phenotypic effects of a loss-of-function of the *HPS3* gene in French Bulldogs with the cocoa coat color. This study reports the resulting coat colors when the cocoa allele is combined with mutant alleles at other coat color loci and the effects of *HPS3* deficiency on platelet morphology and blood coagulation parameters [12].

*Monogenic Inherited Diseases*

With a total of nine articles, this is the largest section of this Special Issue. All of them report causal or highly plausible candidate causal variants. Many of them provide the first clinical and pathological descriptions of these single gene disorders in domestic animals. Two studies report causal variants for X-linked recessive blood coagulation disorders: an *F8* variant was found in Rhodesian Ridgebacks with hemophilia A [13], and an *F9* variant was found in mixed-breed dogs with hemophilia B [14]. A *FYCO1* variant was identified in Wirehaired Pointing Griffon dogs with juvenile cataracts [15]. Three studies identify single gene defects leading to syndromic phenotypes. A *MYO5A* variant is reported in a single Miniature Dachshund with coat color dilution and a lethal neurological defect resembling the human Griscelli syndrome type I [16]. A new late-onset progressive retinal atrophy (PRA) in Shetland Sheepdogs that is associated with an upturned nose and coat and tooth defects was phenotypically characterized and a variant in *BBS2* unraveled as the likely sole causal variant [17]. Another syndrome in Cane Corso dogs, termed the dental-skeletal-retinal anomaly, was phenotypically characterized and a causal variant in *MIA3* was identified that is hypothesized to cause a defect in cellular export in the post-translational maturation of various collagens [18]. A new *PRKG2*-associated skeletal dysplasia leading to pronounced disproportionate dwarfism is reported in Dogo Argentino dogs [19]. A *LAMA2* variant was identified as the most likely cause for congenital muscular dystrophy in an Italian Greyhound [20]. Finally, Garcia et al. report the third known pathogenic *COL7A1* variant in dogs leading to a dystrophic epidermolysis bullosa in Basset Hounds [21].

*Complex Diseases*

Five articles in this Special Issue shed new insights into complex diseases in dogs. A global RNA-seq experiment revealed transcriptomic changes in the skin of alopecic Bohemian Wire-haired Pointing Griffon dogs [22]. Whole genome sequencing experiments enabled the identification of new potential risk variants for symmetrical lupoid onychodystrophy (SLO) in Bearded collies [23]. Norton et al. report the first heritability estimate and GWAS for episodic exercise-induced collapse in Border Collies [24]. Genetic risk factors for deafness were investigated in Australian Stumpy Tail Cattle Dogs [25]. Many giant dog breeds are highly predisposed to develop osteosarcoma. Letko et al. report on a large GWAS in Leonberger dogs replicating the strong effect of the CDKN2A/B risk locus that was previously identified in other breeds [26].

*Genetic Diversity*

The Norwegian Lundehund breed is characterized by extreme inbreeding and several disease predispositions that are hypothesized to result from inbreeding depression. Melis et al. report on a genetic rescue experiment that uses introgression of the phenotypically similar Norwegian Buhund in an attempt to increase genetic diversity in the Norwegian Lundehund [27]. The African painted dog (*Lycaon pictus*) is a highly endangered wild canid species. Miller-Butterworth et al. investigated the genetic diversity in the captive population of North American zoos to provide a basis for the future breeding management and conservation of this iconic species [28].

*Ancient DNA*

Finally, the Special Issue “Canine Genetics 2” contains a study on the mitochondrial DNA diversity of six 25,000–45,000-year-old Asiatic wild dogs or dholes (*Cuon alpinus*) whose fossils were found in Europe. These data will help to better understand the migration and decline of the once wide-spread species that is now restricted to South and South-East Asia [29].

*Conclusions*

The 21 articles compiled in this Special Issue nicely represent the breadth of canine genetic research. These research findings advance our basic understanding of gene functions, genetic diversity and evolution. Many studies enable direct applications, such as the introduction of genetic testing or improved breeding management, that will foster the health and wellbeing of dogs and wild canids.
